# Natural biowaste material-based green triboelectric nanogenerators for self-powered gait monitoring

**DOI:** 10.1039/d5ra09038e

**Published:** 2026-01-14

**Authors:** Md. Mahmodul Hasan, Nasrin Sultana Ety, Fakhrul Islam, Subrata Bhowmik, Kamaruzzaman, S M Sohel Rana

**Affiliations:** a Department of Electrical and Electronic Engineering, Noakhali Science and Technology University Noakhali Bangladesh smsr.aece@gmail.com; b Center for Energy and Sensor Innovation, Noakhali Science and Technology University Noakhali Bangladesh

## Abstract

The advancement of triboelectric nanogenerators (TENGs) has attracted increasing interest in recent years due to the growing tension between the increasing demand for energy and the increasing destruction of fossil fuels. For environmentally friendly and self-sufficient devices with high outputs, a TENG based on natural biomaterials is essential. Herein, we report natural biowaste material-based green triboelectric nanogenerators for self-powered gait monitoring. We systematically investigated three novel tribonegative materials: elephant apple, turmeric peel, and taro stem powder. The elephant apple powder-based TENG (EA-TENG) provides the highest output voltage because of its fibrous surface structure. The EA-TENG exhibits an open circuit voltage (*V*_oc_) of 304 V, yielding a maximum power density of approximately 6.4 W m^−2^, high stability, achieving a high sensitivity of 2.78 V kPa^−1^, and operates in the pressure range of 1–126 kPa. Finally, we showcase its potential by integrating the EA-TENG into flexible sensors capable of accurately monitoring human movements, with a motion detection accuracy of 99.3% achieved using a deep learning-based model. This study comprehensively examines the fundamental triboelectric characteristics of natural fibers and the influence of material composition on output performance, thereby contributing to the field of sustainable energy harvesting technologies and green self-powered sensor applications to reduce electronic waste.

## Abstract 2

We developed an elephant apple powder-based biodegradable TENG from natural biowaste materials for self-powered gait monitoring to reduce electronic waste.

## Graphical abstract

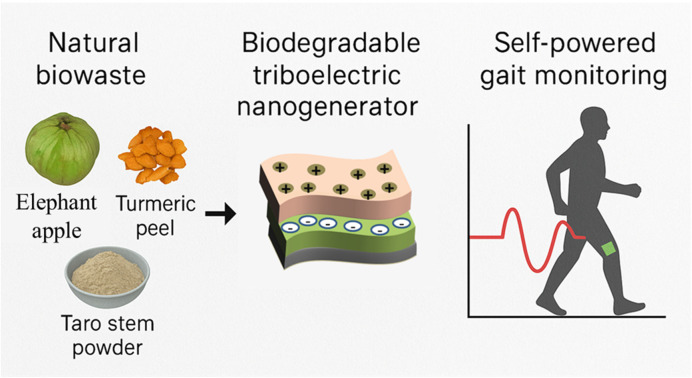

## 1. Introduction

The rapid advancement of the Internet of Things (IoT) has generated significant interest in triboelectric nanogenerators (TENGs) due to their ability to capture energy by converting excess mechanical energy from the environment into sustainable electrical energy.^1–3^ Unlike conventional electric generators, TENGs operate without the need for limited fossil fuels and offers advantages such as higher output performance, greater energy conversion efficiency, low cost, lightweight design, and a wide variety of material options.^4,5^ As a result, the extraction of biomechanical energy from human movement has received significant attention over the past decade due to its potential applications in recharging batteries for portable electronic devices and powering autonomous sensor systems.^6–8^ By combining contact electrification with electrostatic induction, TENGs may be fabricated from various materials with different designs that offer high output power.^9,10^ Recently, low-powered, wearable, and portable electronics like smartwatches and health-monitoring sensors have become increasingly integrated into our daily lives.^11,12^ But the majority of these gadgets are battery-powered, which harms the environment and limits their application in flexible, self-sustaining, and biocompatible electronic systems.^13,14^ Therefore, figuring out a better method to get rid of electrical waste, especially when it comes to disposing of batteries, is highly important. In order to address these issues, various energy harvesters and self-powered sensors utilizing TENG have been developed for a range of specific applications.^15–17^ These include wearable TENG, triboelectric negative ion generators, self-rechargeable cardiac pacemakers, smart insoles, motion monitoring, rehabilitation, healthcare autonomous powering sensor systems.^18–21^ It is also used for specific monitoring, human–machine interfaces (HMI), and disease diagnostics, all aimed at enhancing the quality of human life.^22–24^ Moreover, soft substrates that can be stretched and folded are ideal for creating flexible electronics (FEs).^19^ Despite their promise, many traditional TENGs are constructed using synthetic substrates like textiles, polyimide (PI), hydrogel, polyethylene terephthalate (PET) films, and elastomers like polydimethylsiloxane (PDMS), which are examples of flexible substrates on which electro-conducting components, such as metals, conducting polymers, and carbon-based materials, are commonly built.^5,25–27^ However, the usage of these substrates can lead to the accumulation of electronic trash (e-waste) because many of them are not biodegradable.^28^ As a result, the majority of traditional TENGs, which are constructed from such hostile materials, raise questions about their long-term viability, which could restrict their usage in the future.^29,30^ To address these challenges, researchers are exploring biomaterial-based TENGs because these starting materials are easily extracted from the environment or recovered waste, which not only preserves resources and lowers pollution but also has the benefit of being non-toxic and safe for human health, making them more appropriate for creating wearable electronics.^31^ Natural materials like cellulose, lignin, proteins, and starch have been used to create a range of inexpensive, biodegradable, and environmentally friendly electronic devices in recent years.^32–35^ As a consequence, different biodegradable starches, such as rice sheets, egg whites, chitin, polylactic acid, and silk fibroin, have been utilized to create TENGs, which cause no environmental hazards.^36–38^ Rani *et al.* reported a pioneering approach to sustainable energy generation by repurposing discarded cigarette filters (CFs) and plastic waste as triboelectric layers in a compact TENG device. The authors demonstrate impressive output metrics—42.8 V and a peak power density of 63.2 mW m^−2^—under low-frequency mechanical stimuli, enabling the device to power 44 LEDs and an LCD timer.^39^ Lin *et al.* reported eggshell membrane tribopositive layers, offering high surface charge density and mechanical resilience for wearable sensing.^40^ Pias *et al.* reported water hyacinth root-based TENGs demonstrating power densities up to 5 W m^−2^ and sensitivity of 3.2 V kPa,^1^ with deep learning-enabled motion classification accuracy of 99.3%.^41^ Xia *et al.* reported a tea leaf-based TENG for powering and sensing purposes.^42^ Gao *et al.* introduce a biodegradable triboelectric nanogenerator (PAG-TENG) fabricated from waste bone gelatin modified with POSS polymer, designed to stimulate seed germination through electrostatic fields. When compared to synthetic polymers, these natural materials have the inherent advantages of being readily available, inexpensive, biocompatible, degradable, and easily accessible for chemical modification.^43^ They can be employed as implantable medical diagnostic and healthcare devices in a safe and biocompatible manner.^44^ To meet the e-waste reduction standards, its use may also be devoid of e-waste production. One specific drawback in the early TENG designs is the materials' lack of flexibility, which is necessary for applications in next-generation electronics such as biomechanical monitoring sensors, flexible and touch screen displays, electronic timepieces, and electronic skin.

Herein, this work proposes high-performance triboelectric nanogenerators based on elephant apples that are flexible, environmentally friendly, and biowaste. Thus, the chosen natural materials, turmeric peel, taro stem, and elephant apple, are inexpensive, renewable, and abundantly available, providing a sustainable substitute for their traditional synthetic equivalents. In addition, we optimized the systematic evaluation of their triboelectric performance; TENGs were fabricated and characterized systematically. The optimized elephant apple-based TENG illustrated a maximum open-circuit peak-to-peak voltage and an instantaneous power density among the developed bio polymer-based devices. Finally, a human–machine interaction system was developed, highlighting the sustainable TENGs' encouraging potential for use in wearable self-powered sensors, motion tracking for people, and machine learning technologies. The use of waste material as a renewable resource for green energy harvesting (triboelectric negative) demonstrates potential for sensing applications.

## 2. Experimental section

### 2.1 Materials

Fresh elephant apple, turmeric, and taro were purchased from the local market of Bangladesh. Conductive fabric tape with a contact resistivity of less than 0.1 was acquired from Daraz in Bangladesh.

### 2.2 Preparation of elephant apple/taro stem/turmeric powder

Fresh elephant apples were collected from trees, ensuring that ripe and healthy elephant apples were selected. The elephant apples that were gathered were properly cleaned under running water to get rid of any pollutants, dust, or debris that might have stuck to the surface. The cleaned elephant apples were sliced into thin layers (approximately 2–3 mm thick). Slicing facilitates more efficient drying and uniform moisture removal. These slices were dried for almost 3 days to remove residual moisture, which is essential for improving the triboelectric characteristics. Then, a high-speed blender was used to grind the dried slices into a fine powder. The powder particle size should be tiny enough to offer a maximum surface area for the best triboelectric performance. The sieving technique was utilized for the separation of powders into specified size ranges and for the elimination of larger agglomerates, which facilitates a consistent particle distribution. The prepared elephant apple powder is stored in an airtight container to prevent moisture absorption and contamination. Similar procedures were followed to prepare taro stem and turmeric powder.

### 2.3 Device fabrication

**Fig. 1:**
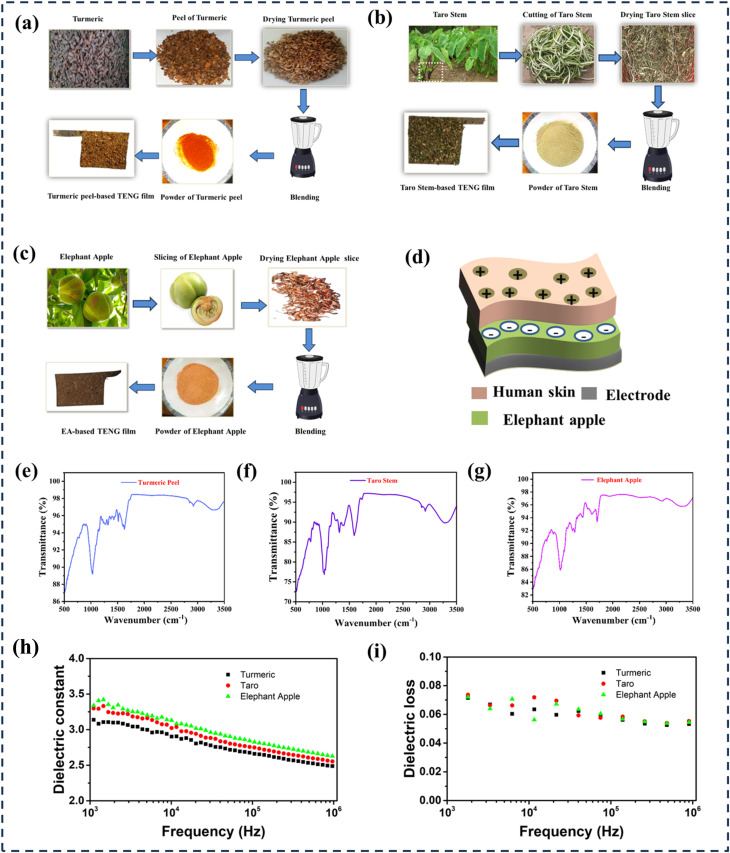
The fabrication process of TENG uses natural materials. (a) Development of TENG based on turmeric peel powder. (b) Development of TENG based on taro stem powder. (c) Development of TENG based on elephant apples powder. (d) Diagrammatic representation of the TENG incorporating elephant apple powder. FTIR spectra of the structures: (f) turmeric peel powder-based TENG, (g) taro stem powder-based TENG, and (e) elephant apple powder-based TENG, respectively. (h and i) Frequency-dependent dielectric constant and dielectric loss of three natural materials, such as turmeric, taro, and elephant apple.

Fig. 1d shows the fabrication of elephant apple-based TENG. A conductive fabric electrode including conductive paste was used. Nylon-coated conductive fabrics exhibit significant electrical conductivity, lightweight characteristics, stability, and reliability. The surface resistivity measured for these electrodes was approximately 0.08 Ω/sq., encompassing those with a low thickness of 0.030 mm. The fabric electrode was cut into a rectangular shape (2.6 cm × 1.9 cm). The dimensions were chosen to ensure a sufficient area of contact between the human skin and the electronegative substance, which could improve the efficiency of charge transfer. Elephant apple powder was attached by conductive adhesive to a fabric electrode that conducts electricity. In order to keep the powder and electrode electrically connected, the adhesive ensured that the powder and electrode were in contact, which was necessary for efficient charge transfer. To ensure consistent performance, the elephant apple powder layer's thickness was kept uniform using a 3D printed template, and 0.25 g of powder was selected for every sample. In order to retain the mechanical integrity of the layer and provide adequate contact area, a thickness of roughly 1 mm was tried to be maintained. To ensure complete coverage and uniformity of the coated layer, fixed-volume casting and repeated coating were employed. The copper wire was connected to a digital oscilloscope through a probe for voltage measurement. Turmeric peel powder-based TENG and taro stem powder-based TENG were fabricated using the same procedure.

### 2.4 Characterization and measurements

The Fourier transform infrared spectrometer (FTIR, SOP Spectrum Two) was employed to assess the transmittance of the fabricated composite film. A scanning electron microscope (SEM,Zeiss EVO MA 15 Scanning Electron Microscope) operating at an accelerating voltage of 10 kV was used to characterize the surface morphology of triboelectric materials. A digital oscilloscope GDS-1102B of GWINSTEK was used with a probe (GTP-100B-4) of 100 MHz/10 MHz to measure the output voltage generated when the electronegative layer was placed on the electrode against human skin. The simulation was conducted by utilizing the COMSOL software suite. Dielectric constant was measured using an LCR meter. A flexi-force sensor (Flexiforce-A201) was used for pressure measurement, interfaced with an Arduino Uno. The sensor was calibrated using known weights to establish a force–voltage relationship, and the Arduino was programmed to convert analog signals into real-time pressure data with noise reduction routines, ensuring accurate and reproducible measurements.

### 2.5 Simulation

The potential distribution of the TENG has been ascertained utilizing the Finite Element Method (FEM) in COMSOL Multiphysics 5.4. The potential distribution was assessed in stationary research employing a 2D axisymmetrical electrostatic module. A TENG model is constructed utilizing air as the surrounding medium within the “geometry” section of this simulation. For simulation, the parameter was employed in the following manner: the dimensions of the two friction layers are 1 mm in height and 25 mm in width. Correspondingly, the friction layer and the electrode exhibit identical dimensions in both width and height. The observed range between the two layers was 0 to 5 mm, which contributed to the frictional forces present. Subsequently, the material properties of the designated portion are established. In the simulations conducted using COMSOL for TENGs, mechanical boundary conditions were established by immobilizing the bottom substrate while implementing a time-dependent displacement on the upper triboelectric layer to replicate the contact-separation motion. The electrical boundary conditions entailed grounding one electrode and applying a time-varying potential to the other, while the insulating surfaces were subjected to floating potential or zero charge conditions. Boundary conditions were established by assigning surface charge densities to the triboelectric layers, with the positively charged material designated at +1.60 × 10^−6^ C m^−2^ and the negatively charged material at −1.60 × 10^−6^ C m^−2^, reflecting the electrostatic potential produced during contact electrification. In the subsequent analysis, the relative permittivity of the positive material is established at 4, while the relative permittivity of the negative material is designated at 3.5, thereby ensuring the requisite triboelectric properties. A physics-controlled mesh with a defined element size was utilized, integrating boundary layer meshing in proximity to dielectric interfaces and electrode surfaces to effectively capture the electric field gradient. The material properties, such as Young's modulus, Poisson's ratio, density, and relative permittivity for each layer, were established based on data sourced from the library and experimental measurements. Ultimately, we commenced the process of employing computational methods to determine the potential distribution in the designated study section.

## 3. Results and discussion

Fig. 1a–c shows the manufacturing process of the turmeric peel-based TENG, taro stem-based TENG, and elephant apple-based TENG. Details are given in the experimental section. Fig. 1d shows the schematic of the TENG that consists of human skin, which is tribo-positive, and elephant apple powder, which is tribo-negative due to its cellulose and phenolic content, and a conductive fabric electrode. The remarkable flexibility of the composite film allows the TENG to attach to the human skin, collect biomechanical energy, and track biomedical data. Fig. 1e–g shows the FTIR spectra patterns of turmeric peel powder, taro stem powder, and elephant apple powder, respectively. The FTIR analysis of turmeric peel powder reveals distinct absorption bands corresponding to its functional groups and biochemical constituents (Fig. 1e). A broad peak observed around 3300 cm^−1^ is attributed to O–H stretching vibrations from phenolic compounds' hydroxyl groups in alcohols, and residual moisture.^45^ Peaks near 2920 cm^−1^ and 2850 cm^−1^ arise from asymmetric and symmetric C–H stretching in aliphatic chains, likely associated with fatty acids or structural polysaccharides. A sharp band at 1730 cm^−1^ indicates the presence of C=O stretching vibrations, characteristic of ester or ketone groups, possibly from curcumin derivatives or plant cell wall components. The region between 1600–1500 cm^−1^ shows aromatic C=C stretching and conjugated carbonyl groups, consistent with the benzene rings in curcuminoids. Peaks in the range 1450–1375 cm^−1^ correspond to C–H bending modes in methyl (–CH_3_) and methylene (–CH_2_) groups. Strong absorptions between 1260–1000 cm^−1^ are assigned to C–O stretching in cellulose, hemicellulose, and lignin, highlighting the fibrous lignocellulosic structure of the peel. Notably, the absence of prominent N–H stretches (∼3300 cm^−1^) or amide bands (∼1650 cm^−1^) suggests minimal protein content. The FTIR spectrum of taro stem powder shows distinct absorption bands characteristic of its biochemical composition (Fig. 1f). Elephant apple powder's FTIR spectrum (Fig. 1g) displays different absorption bands that are indicative of its biological makeup. A wide drop at around 3300 cm^−1^ is indicative of O–H stretching vibrations, which are probably caused by moisture, alcohols, or phenolic chemicals. Peaks in the ∼2900 cm^−1^ area, which are characteristic of polysaccharides or lipids, show C–H stretching in aliphatic chains. A drop at around 1700 cm^−1^ indicates C=O stretching, which might be caused by carboxylic acids, ketones, or esters—all of which are often found in secondary metabolites generated from plants. The 1500–1600 cm^−1^ range could include conjugated carbonyl groups or aromatic C=C bonds linked to flavonoids or phenolic acids. The fibrous polysaccharide content is highlighted by strong absorption below 1300 cm^−1^ (peaking about 1000–1200 cm^−1^) caused by C–O–C and C–O stretching in cellulose, hemicellulose, or lignin. The lack of noticeable N–H bands (around 3300 cm^−1^ for amines) indicates that there is not much protein present.

**Fig. 2:**
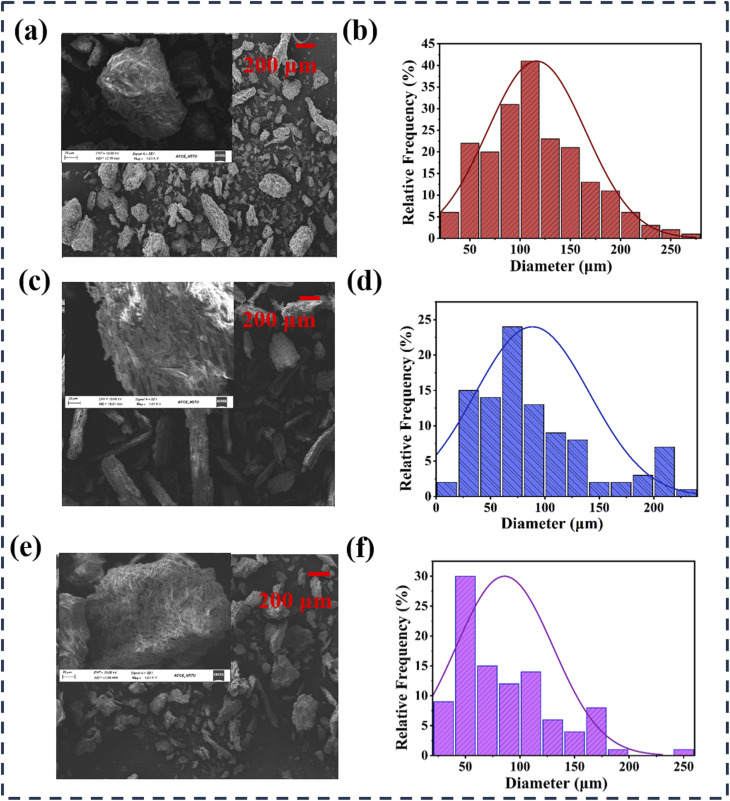
A comprehensive analysis of the natural elements' morphology used in the TENG. (a) SEM image of turmeric peel powder. Inset: magnification view of the SEM image. (b) Diameter distribution of turmeric peel powder's particles. (c) SEM image of taro stem powder. Inset: magnification view of the SEM image. (d) Diameter distribution of taro stem powder particles. (e) SEM image of elephant apple powder. Inset: magnification view of the SEM image. (f) Diameter distribution of elephant apple powder's particles.

Fig. 1h illustrates the frequency-dependent dielectric constant of three natural materials, such as turmeric, taro, and elephant apple, within the frequency range of 10^3^ to 10^6^ Hz. The elephant apple exhibits a dielectric constant of 3.5, the highest among the materials investigated, followed by taro at 3.3 and turmeric at 3.0. These values indicate a greater polarizability, which may enhance the potential applications in TENG. Fig. 1i presents the variation of dielectric loss as a function of frequency for turmeric, taro, and elephant apple across the range of 10^3^ to 10^6^ Hz. The elephant apple exhibits the highest dielectric loss, followed by taro and turmeric, indicating a more significant polarization lag and internal friction within its molecular architecture. The morphology of particles in selected materials was evaluated in Fig. 2 for their impact on TENG performance. Additionally, the performance of the TENG is influenced by the contact area between the frictional materials. Furthermore, the inner gaps of the triboelectric layers, such as porosity, positively affect the output performance of the TENG. Fig. 2a displays the SEM image of powdered turmeric peel, which shows a range of particle sizes with uneven edges, random surface textures, and porosity. The distribution of particle diameter is shown in Fig. 2b. The histogram shows that the particle diameter ranges between 100 and 120 µm, with an average diameter of around ∼116.3 µm and a probability of about 41%. Furthermore, because of the characteristics mentioned above, it has a large surface area and generates more electrostatic charges due to the triboelectric effect. The SEM image of the taro stem particles is displayed in Fig. 2c, while the particle diameter distribution histogram is illustrated in Fig. 2d. The resulting particles, as shown in the SEM image, are distributed randomly and free of defects and beads. Furthermore, this material also has an irregular surface structure. With fiber sizes ranging from 3 to 240 µm, the histogram displays a normal distribution. With a likelihood of roughly 24%, the fiber diameter distribution lies between 60 and 80 µm with an average diameter of ∼88.49 µm. Fig. 2e illustrates the typical morphological profile of the elephant apple, which has a lateral size between 5 and 260 µm and a well-defined microstructure. This material demonstrates a larger surface area, superior particle uniformity, and significant porosity. These characteristics result in a greater generation of electrostatic charges through the triboelectric effect compared to the other two materials. Fig. 2f displays the particle diameter distribution. The distribution shows that almost 30% of the particles, with an average diameter of ∼85.86 µm, are in the 40–60 µm range.

**Fig. 3:**
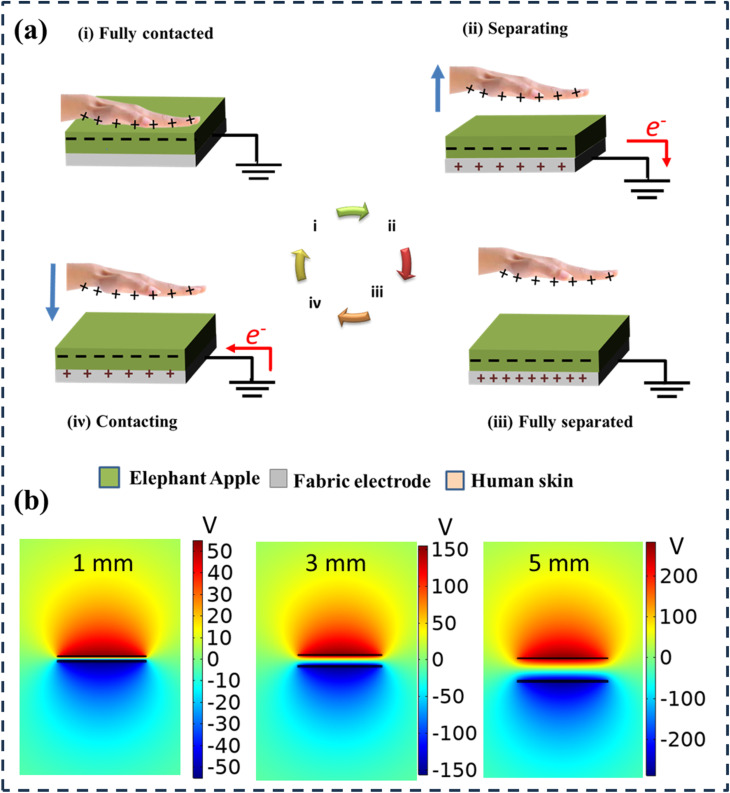
(a) Schematic illustration of operational principles of TENGs utilizing elephant apple powder. (b) COMSOL-based simulation for the numerical modeling of the potential distribution of TENG at different separation distances between the human skin surface and the elephant apple powder.

Fig. 3a depicts the operational principle of the TENG, which is constructed using friction layers made from human skin and EA powder. In the contact phase, the application of mechanical pressure facilitates direct interaction between the human skin and the EA powder layers, thereby inducing triboelectric charging. Human skin develops a net positive surface charge as a result of its unique electron affinities, whereas the EA powder gathers a corresponding negative charge. When the two layers are separated, an electrostatic potential difference is generated between the conductive fabric electrodes incorporated within the device. The potential difference facilitates the transfer of electrons through the external circuit by means of electrostatic induction. Electrons migrate from the negatively charged electrode associated with the EA powder to the ground, thereby balancing the induced charges. Upon achieving electrostatic equilibrium, the flow of current comes to a stop. In contrast, during the following compressive phase, the diminished interlayer distance alters the potential gradient, resulting in electron flow in the reverse direction (from ground to electrode). The periodic motion of contact and separation among the friction layers, combined with the dynamics of triboelectric charging and electrostatic induction, produces a sustained alternating current within the external circuit. This mechanism facilitates the conversion of repetitive mechanical energy into a usable electrical output by the TENG.

As illustrated in Fig. 3a, a basic mechanical setup was used to press and distance the human skin from the elephant apple layer. To ensure uniform contact and optimize charge transfer, the elephant apple layer was pressed against the skin while keeping constant force on the electrode. The triboelectric effect caused electrons to move from the human skin to the elephant apple layer during contact. Contact was then instantly released to balance this potential imbalance, which resulted in the creation of a charge that was transferred through the electrode as an electric current. The separation speed affects the rate at which possible disparities are formed. In general, a higher instantaneous current is the result of faster separation. The simulation results of the electrical potential distribution in the elephant apple-based TENGs are shown in Fig. 3b when the human skin is positioned at three distinct points (1, 3, and 5 mm, respectively). It is discovered that the electrical potential increases linearly when the hand is in the middle of the position. Both surfaces experience a zero electric potential when the human skin is completely in touch with the elephant apple film surface. When they are separated by 6 mm, the electric potential on the hand surface rises to 304 V.

**Fig. 4:**
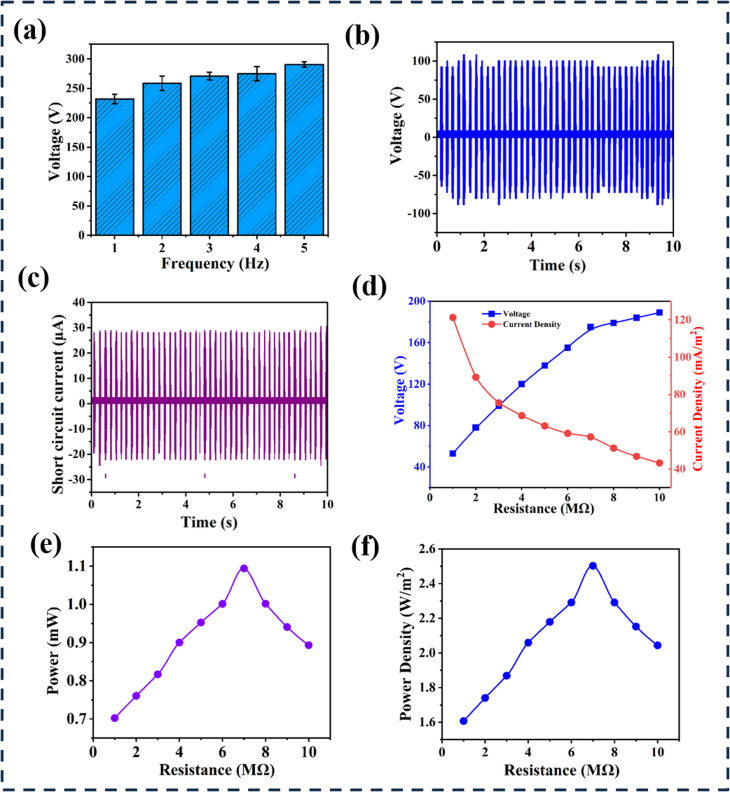
Illustration of turmeric peel powder-based TENG's output (a) voltage variations over frequency of turmeric peel-based TENG. (b) Comparison of output voltages of turmeric peel powder-based TENG with respect to time. (c) Comparison of the output current of turmeric peel powder-based TENG with respect to time. (d) Variations of output voltage and current of turmeric peel powder-based TENG with varying resistance. (e) Variations of output power with varying resistance. (f) Variations of power density with varying resistance.

The output voltage of the turmeric peel-based TENG was observed at different frequencies. Fig. 4a shows that the output voltage rises progressively with frequency. The results demonstrate a progressive increase in output voltage with rising frequency, highlighting the device's responsiveness to mechanical input dynamics. At lower frequencies, the voltage output is modest due to fewer contact-separation cycles per unit time, limiting charge accumulation. As the frequency escalates, the rapid cycling enhances charge generation and transfer efficiency, driven by the repeated mechanical interaction between the tribo-negative turmeric peel layer and the tribo-positive counter-material. To assess measurement reliability, output voltage was recorded three times at specific frequencies, and standard deviation was applied to quantify variability. The error bars indicate the consistency of output voltage, with larger bars reflecting greater fluctuations and smaller bars denoting more stable values. These findings collectively underscore the TENG's potential for efficient energy harvesting and the critical role of resistance and frequency optimization in enhancing performance, paralleling the results observed in the turmeric peel-based TENG. The output characteristics of the turmeric peel-based TENG were systematically evaluated, revealing a peak open circuit voltage of 196 V and a current density of 121 mA m^−2^, as shown in Fig. 4b and c. The output voltage increased with external resistance, reaching a maximum of 189 V at 10 M Ω, while current decreased proportionately, indicating an inverse relationship consistent with Ohm's law Fig. 4d. Furthermore, the power output peaked at approximately 1.09 mW with a voltage of 175 V at 10 M Ω, demonstrating the maximum power transfer theorem, where power initially increased with resistance, peaking at around 7 M Ω before declining Fig. 4e. These findings highlight the effective energy harvesting capabilities of the turmeric peel-based TENG and the importance of optimizing resistance for enhanced power output. Fig. 4f illustrates that the TENG achieved a maximum power density of approximately 2.50 W m^−2^ at an external resistance of 7 M Ω, with the electrode area containing turmeric peel powder measuring 4.37 cm^2^.

**Fig. 5:**
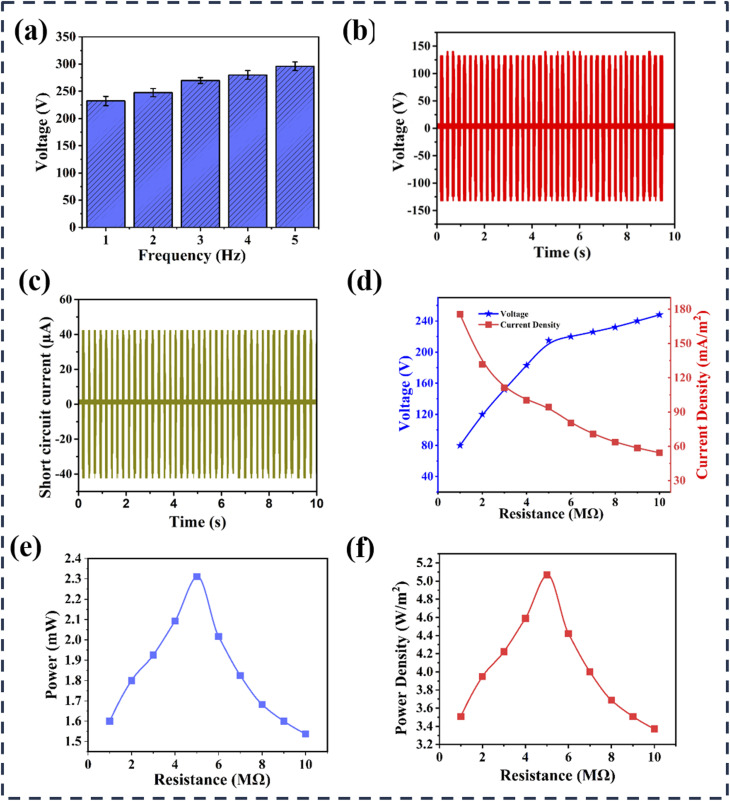
Illustration of taro stem powder-based TENG's output (a) voltage variations over frequency of taro stem-based TENG. (b) Comparison of output voltages of taro stem powder-based TENG with respect to time. (c) Comparison of the output current of taro stem powder-based TENG with respect to time. (d) Variations of output voltage and current of the taro stem powder-based TENG with varying resistance. (e) Variations of output power with varying resistance. (f) Variations of power density with varying resistance.

The output characteristics of the taro stem-based TENG are presented in Fig. 5. Fig. 5a illustrates how the output voltage increases gradually as frequency increases. Three recordings of the output voltage at particular frequencies were made to evaluate the measurement's dependability, and the standard deviation was used to determine variability. Fig. 5b illustrates the output voltage signal with the TENG generating a peak voltage of 280 V. The current density, depicted in Fig. 5c, reached 175 mA m^−2^, indicating robust charge generation capabilities. As shown in Fig. 5d, varying the external resistance resulted in increasing output voltages, peaking at 248 V with a resistance of 10 M Ω, while current exhibited a corresponding decrease with increasing resistance. Fig. 5e highlights the change in power as resistance varies, with a maximum power output of 2.31 mW achieved at 5 M Ω. This behavior aligns with the maximum power transfer theory, where power peaks at 5 M Ω before declining. In Fig. 5f, a peak power density of approximately 5.068 W m^−2^ was attained at 5 M Ω with an electrode area of 4.56 cm^2^ containing taro stem powder. The graph indicates that power density increases with resistance until reaching a maximum, after which it declines. These findings collectively underscore the TENG's potential for efficient energy harvesting and the critical role of resistance and frequency optimization in enhancing performance, paralleling the results observed in the turmeric peel-based TENG.

**Fig. 6:**
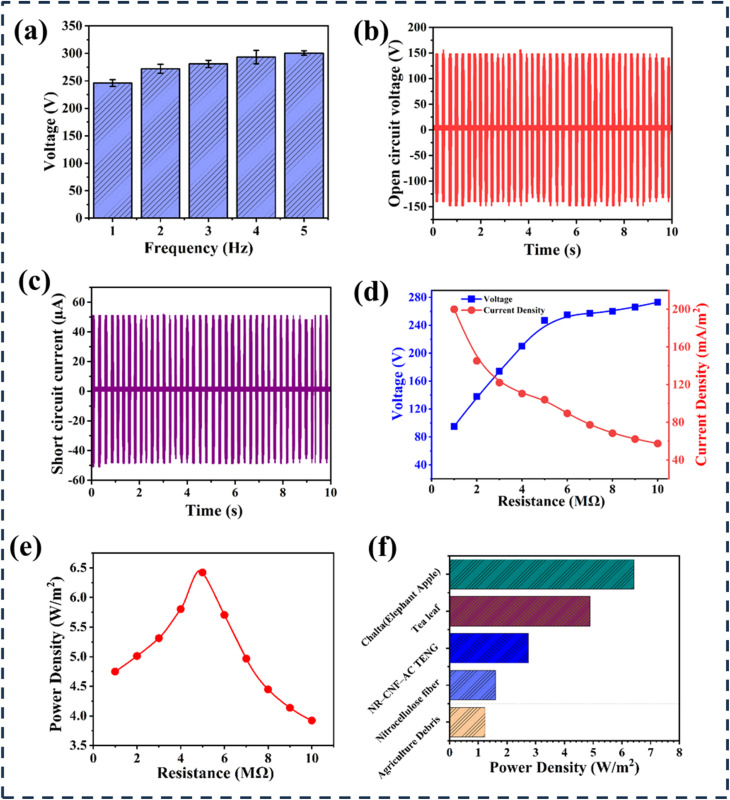
Illustration of elephant apple powder-based TENG's output. (a) Voltage variations over frequency of elephant apple powder-based TENG. (b) Comparison of the output voltages of elephant apple powder-based TENG over time. (c) Comparison of the output current of elephant apple powder-based TENG over time. (d) Variations of output voltage and current of elephant apple powder-based TENG with varying resistance. (e) Variations of power density with varying resistance. (f) Comparison of power density among different natural materials based on TENG.^35,38,42^

The EA-TENG output characteristics are shown in Fig. 6. Fig. 6a depicts that the output voltage rises steadily with increasing frequency. Fig. 6b illustrates that the TENG's voltage was 304 V, and Fig. 6c shows it current density was 200 mA m^−2^. Fig. 6d illustrates how changing the external resistance raised output voltages, which peaked at 273 V with a resistance of 10 M Ω while decreasing current. According to the maximum power transfer theory, it states that power peaks at this resistance before decreasing. Fig. 6e illustrates the variations in power density as resistance is adjusted, showing a maximum power density of approximately 6.4 W m^−2^ at 5 M Ω, with the electrode area containing taro stem powder measuring 4.75 cm^2^. The graph indicates that power density increases with added resistance until reaching a peak, after which it declines. Fig. 6f demonstrates the comparison of different natural element-based TENG according to their power density. Here, EA-based TENG has a power density of 6.4 W m^−2^, comparatively larger than tea leaf-based TENG (4.89 W m^−2^), NR–CNF–AC TENG (2.74 W m^−2^), and so on. The maximum power density of EA-based TENG outperformed (6.4 W m^−2^) and turmeric peel (2.50 W m^−2^). This implies that EA-based TENGs are more suited for small-scale energy harvesting applications since they can produce more energy per unit area. The output performance comparison of biomaterial-based TENG is shown in Table 1.

**Table 1:** Output performance comparison of bio materials based TENG

Tribo material	Feature	Voltage	Power density	Applications	References
Cellulose/PET	Biodegradable	55.8 V	29 mW cm^−2^	Self-powered sensing	46
Fish bladder/FEP	Biodegradable	106 V	200 µW cm^−2^	Electronic skin	47
Chitosan–diatom/FEP	Biological safety	150 V	15.7 µW cm^−2^	Motion sensor	48
Silk/PET	Biodegradable	268 V	193.6 µW cm^−2^	Powering a microsensor	49
Egg film/PTFE	Biodegradable	101.5 V	—	Power source	50
Balsa wood/PTFE	Biodegradable	81 V	57 µW cm^−2^	Power source	51
Inner surface of onion/Outer surface of onion	Biodegradable	32 V	1.27 W m^−2^	Self-powered sensor	52
Celery cabbage/Bread	Biodegradable	15 V	1.0 mW m^−2^	Energy harvesting	53
Rose petal/PMMA	—	30.6 V	27.2 mW m^−2^	Energy harvesting	54
Silk/Rice paper	Biocompatible	34 V	21.6 mW m^−2^	Energy harvesting and sensor	55
Rice paper/PVC	Recyclable	244 V	37.64 µW cm^−2^	Energy harvesting	56
Human skin/Elephant apple powder	Biodegradable	304 V	6.4 W m^−2^	Self-powered sensor	This work

**Fig. 7:**
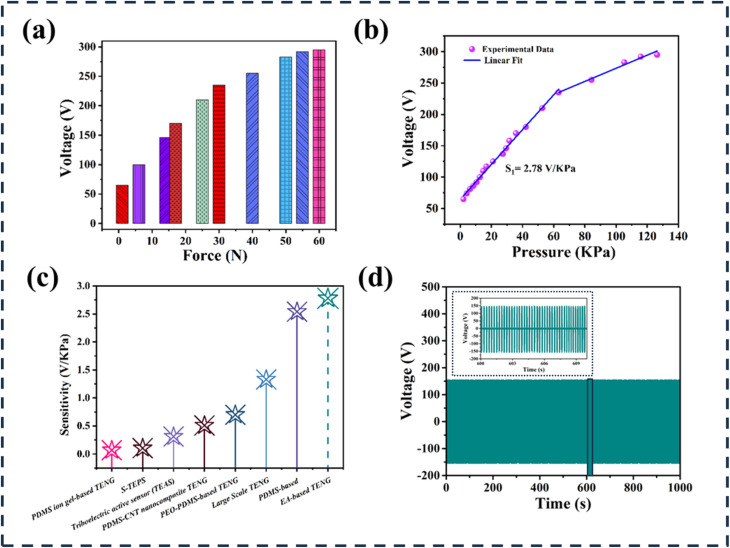
(a) Output voltage based on changing compressive forces ranging from 0 to 60 N. (b) The relationship between the input pressure, ranging from 0 to 140 kPa, and the TENG's output voltage. (c) Assessment of the developed TENG's sensitivity compared to previously published TENGs.^43,57–63^ (d) Stability evaluations for 3000 continuous cycles.

The performance of the EA-TENG was rigorously evaluated under varying compressive forces to assess its pressure sensitivity and operational stability. As depicted in Fig. 7a, the output voltage of the EA-TENG exhibits a direct proportionality to the applied compressive force, reaching its peak value at maximum force. This behavior is attributed to the increased contact area between the tribo-positive human skin and the tribo-negative elephant apple powder layer under higher pressures. A larger contact area enhances interfacial electron transfer, thereby amplifying surface charge density and improving output performance. Fig. 7b quantifies the relationship between input pressure (0–126 kPa) and output voltage. The EA-TENG demonstrates a linear response in the low-pressure regime (0–60 kPa) with a sensitivity of 2.78 V kPa^−1^, a critical metric for detecting subtle biomechanical motions. Beyond 60 kPa, the voltage continues to rise but deviates from linearity, eventually saturating at pressures exceeding 105 kPa. This saturation likely arises from physical limitations in material deformation, where further increases in pressure no longer expand the effective contact area, capping charge density. A comparative sensitivity analysis (Fig. 7c) highlights the EA-TENG's superiority over previously reported triboelectric devices, particularly in low-to-moderate pressure ranges. This enhanced performance stems from the optimized triboelectric pair (skin and cellulose-rich elephant apple powder) and the device's structural design, which maximizes charge generation efficiency. Long-term durability was assessed through 3000 contact-separation cycles at 3 Hz (Fig. 7d). The EA-TENG maintained stable output voltage with negligible degradation, underscoring its mechanical robustness and material resilience. This stability is crucial for applications that require sustained operation, such as continuous health monitoring or energy harvesting from repetitive movements.

**Fig. 8:**
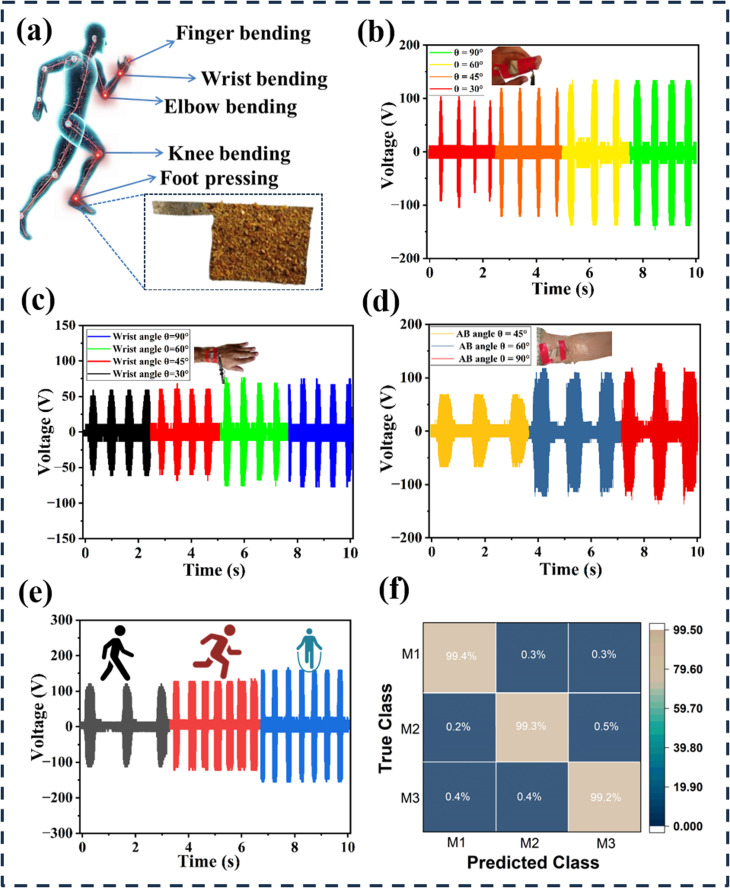
(a) Schematic representation of a human model including TENGs for continuously monitoring movement in humans. Generated voltage signals of the self-powered sensor with the integrated elephant apple-based TENG in response to (b) bending of the finger, (c) bending of the wrist, (d) bending of the elbow, and (e) foot pressing. (f) Confusion matrix for gait analysis.

The EA-TENG exemplifies a groundbreaking advancement in wearable electronics, prioritizing longevity and flexibility—key attributes for seamless integration into daily wear and sustained functionality. As illustrated in Fig. 8a, the device operates as a self-sustaining bio-motion sensor, leveraging the triboelectric effect to convert mechanical energy from human movement into electrical signals. This capability enables real-time monitoring of physiological activities, offering valuable biofeedback for applications in healthcare, rehabilitation, and sports science. When the EA-TENG is attached to a finger joint (Fig. 8b), the EA-TENG demonstrates a linear relationship between voltage output and bending angle. As the joint flexes, the increased contact pressure between the tribo-positive skin and the tribo-negative elephant apple powder layer enhances electron transfer, amplifying the output signal proportionally. This precise correlation allows for a quantitative assessment of joint mobility, critical for tracking conditions like arthritis or post-surgical recovery. Further applications include placement on the wrist and elbow joints (Fig. 8c and d), where the sensor captures dynamic motions such as flexion and extension. The device's pliable structure ensures conformal contact with curved surfaces, maintaining signal fidelity even during rapid or repetitive movements. High sensitivity enables the detection of subtle physiological signals (*e.g.*, pulse waves) alongside gross motor activities, highlighting its dual utility in monitoring both cardiovascular metrics and musculoskeletal dynamics. For large-scale motion tracking, the EA-TENG is integrated into footwear (Fig. 8e), where it harvests energy from foot strikes during walking, running, or jumping. The mechanical energy from heel impact drives charge generation, powering the sensor autonomously while simultaneously recording activity patterns. Signal amplitude and frequency variations distinguish between gait phases, enabling detailed biomechanical analysis. This real-time data acquisition is pivotal for applications in athletic performance optimization or fall detection in elderly populations.

Gait analysis serves as a critical diagnostic and rehabilitative tool in sports and medicine, offering insights into psychological well-being and physical recovery. The EA-TENG addresses challenges in gait rehabilitation by functioning as a self-powered biomotion sensor. To validate its utility, a prototype was integrated into the lower shaft of a walking cane, where mechanical interactions between the natural composite films—actuated by ambulatory motion—generated measurable electrical signals. This design enables real-time, non-invasive monitoring of step patterns for elderly or visually impaired users, crucial for detecting irregularities and preventing falls. Laboratory trials demonstrated the sensor's ability to reliably capture walking dynamics, with the TENG's output correlating to step frequency and force. Leveraging a deep learning framework trained on pre-collected gait data, the system achieved 99.3% accuracy in identifying users' motion states (*e.g.*, normal *vs.* irregular steps) while preserving anonymity, as evidenced by a confusion matrix (rows: true classes; columns: predictions) showing <1% misclassification across categories (M1: 99.4%, M2: 99.3%, M3: 99.2%) is shown in Fig. 8f. To achieve 99.3% classification accuracy in gait recognition from EA-TENG output signals, a deep learning model based on a convolutional neural network (CNN) architecture was developed. The confusion matrix (Fig. 8f), was a supervised classification model trained on a balanced dataset comprising approximately 3392 samples across three classes (M1, M2, and M3). The model includes three convolutional layers with ReLU activation functions and max-pooling operations for feature extraction, followed by fully connected layers culminating in a softmax output for three motion classes (M1, M2, M3). The dataset was split using an 80 : 20 train-validation ratio, and performance was further validated using 5-fold cross-validation to ensure robust generalization. As shown in the confusion matrix, the model demonstrates consistent precision across all classes, confirming its reliability for wearable bio-motion sensing. The integration of eco-friendly materials, high sensitivity, and AI-driven analytics positions this TENG as a sustainable, privacy-conscious solution for personalized sports rehabilitation and assistive mobility technologies.

## 4. Conclusion

In this study, a flexible TENG was fabricated using an eco-friendly elephant apple as the electron acceptor layer for self-power sensing and biomechanical energy harvesting. Owing to the inherent attributes of elephant apple, like porosity and enhanced surface roughness, the EA-TENG effectively extracted biomechanical energy from human movements, achieving a peak power density of 6.4 W m^−2^ at 5 M Ω and sensitivity of 2.78 V kPa^−1^, useful to power low-energy electronic devices. In addition, this self-powered wearable TENG system exhibits promising potential for real-time, noninvasive monitoring of bio-motion and physiological signals. Ultimately, we demonstrate its capabilities by incorporating the EA-TENG onto flexible sensors that can precisely track human motions, achieving a motion detection accuracy of 99.3% using a deep learning-based model. This research will facilitate the development of eco-friendly natural biomaterials for wearable TENGs and their potential uses in multifunctional self-powered sensing.

## Author contributions

Md. Mahmodul Hasan: conceptualization, data curation, methodology, visualization, writing – original draft; Nasrin Sultana Ety: conceptualization, data curation, methodology, visualization, writing – original draft; Fakhrul Islam: investigation & methodology, formal analysis, review & editing; Subrata Bhowmik and Kamaruzzaman: resource, formal analysis, funding, review & editing; S M Sohel Rana: conceptualization, methodology, simulation, supervision, resource, funding, formal analysis, review & editing.

## Conflicts of interest

There are no conflicts to declare.

## Data Availability

The data supporting the results of this study are included in the article. Additional raw datasets generated during this research are available from the corresponding author upon reasonable request.
